# 2-Hydr­oxy-1-methoxyxanthen-9-one monohydrate

**DOI:** 10.1107/S1600536809039348

**Published:** 2009-10-23

**Authors:** Guangying Chen, Jun Zhao, Changchun Cen, Changri Han, Xinming Song

**Affiliations:** a Hainan Provincial Key Laboratory of Tropical Pharmaceutical Herb Chemistry, College of Chemistry & Chemical Engineering, Hainan Normal University, Haikou 571158, People’s Republic of China

## Abstract

In the title compound, C_14_H_10_O_4_·H_2_O, isolated from the roots of *Calophyllum membranaceum*, the xanthene ring system is almost planar (r.m.s. deviation = 0.008 Å). In the crystal structure, inter­molecular O—H⋯O and O—H⋯(O,O) hydrogen bonds connect the mol­ecules.

## Related literature

For medicinal and botanical background, see: Zou *et al.* (2005 ▶); Chen *et al.* (2008 ▶).
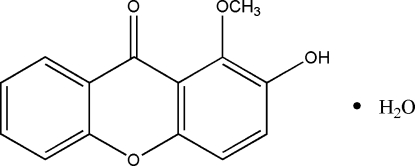


## Experimental

### 

#### Crystal data

C_14_H_10_O_4_·H_2_O
*M* *_r_* = 260.24
Monoclinic, 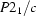

*a* = 8.8008 (6) Å
*b* = 7.0856 (4) Å
*c* = 19.4596 (9) Å
β = 102.402 (4)°
*V* = 1185.16 (12) Å^3^
*Z* = 4
Mo *K*α radiation
μ = 0.11 mm^−1^
*T* = 295 K
0.38 × 0.26 × 0.24 mm

#### Data collection

Bruker SMART CCD diffractometer
Absorption correction: multi-scan (*SADABS*; Bruker, 1997 ▶) *T*_min_ = 0.959, *T*_max_ = 0.974
8151 measured reflections
2919 independent reflections
2269 reflections with *I* > 2σ(*I*)
*R*_int_ = 0.080

#### Refinement

*R*[*F* ^2^ > 2σ(*F* ^2^)] = 0.053
*wR*(*F* ^2^) = 0.164
*S* = 1.04
2919 reflections
181 parameters
H atoms treated by a mixture of independent and constrained refinement
Δρ_max_ = 0.26 e Å^−3^
Δρ_min_ = −0.36 e Å^−3^

### 

Data collection: *SMART* (Bruker, 1997 ▶); cell refinement: *SAINT* (Bruker, 1997 ▶); data reduction: *SAINT*; program(s) used to solve structure: *SHELXS97* (Sheldrick, 2008 ▶); program(s) used to refine structure: *SHELXL97* (Sheldrick, 2008 ▶); molecular graphics: *SHELXTL* (Sheldrick, 2008 ▶); software used to prepare material for publication: *SHELXTL*.

## Supplementary Material

Crystal structure: contains datablocks global, I. DOI: 10.1107/S1600536809039348/hb5095sup1.cif

Structure factors: contains datablocks . DOI: 10.1107/S1600536809039348/hb5095Isup2.hkl

Additional supplementary materials:  crystallographic information; 3D view; checkCIF report

## Figures and Tables

**Table 1:** Hydrogen-bond geometry (Å, °)

*D*—H⋯*A*	*D*—H	H⋯*A*	*D*⋯*A*	*D*—H⋯*A*
O4—H4*A*⋯O1*W*	0.82	1.90	2.7126 (16)	174
O1*W*—H1*A*⋯O2^i^	0.83 (3)	2.03 (3)	2.857 (2)	174 (3)
O1*W*—H1*B*⋯O4^ii^	0.81 (3)	2.34 (3)	2.9540 (19)	134 (2)
O1*W*—H1*B*⋯O3^ii^	0.81 (3)	2.37 (3)	3.1195 (17)	155 (2)

Symmetry codes: (i) 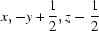; (ii) 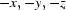.

**Fig. 1.**
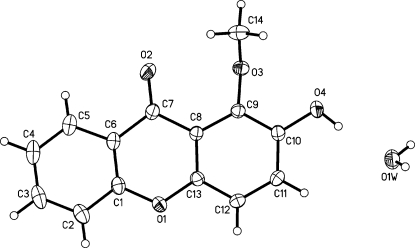
View of (I): displacement ellipsoids are drawn at the 30% probability level.

**Fig. 2.**
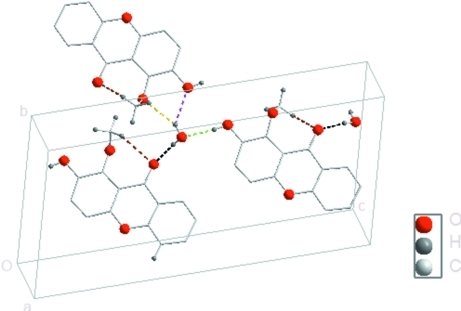
A view of the molecular packing. Dashed lines indicate hydrogen bonds.

## supplementary crystallographic information

### Comment

Secondary metabolites in the plants of Calophyllum membranaceum are mainly xanthones, coumarins and flavonids (Zou *et al.*, 2005; Chen *et al.*, 2008). The plants in this family were used in folk medicine such as, for rheumatism, arthritis, lumbago and wounds (Zou *et al.*, 2005). The title xanthones was isolated from the 75% EtOH extract of the roots of Calophyllum membranaceum which were collected from Lingshui County, Hainan Province, P. *R*. China. We have undertaken the X-ray crystal structure analysis of the title xanthone in order to establish its molecular structure and relative stereochemistry.

The xanthene ring system of (I) (C1-C13/O1) is almost planar, with all atoms lying within 0.008 (8)Å of the mean plane.

In the crystal, molecules are linked by intermolecular O–H···O hydrogen bonds into chains (Fig.2). The hydrogen bonds and angles are listed in Table 1.

### Experimental

Air-dried roots of Calophyllum membranaceum (15.00 kg) were ground and percolated (3 × 2.5 h) with 75% EtOH at 333 K, which was suspended in 1.5 l water and then partitioned with petroleum ether, chloroform, ethyl acetate and n-hexane, successively, yielding a petroleum ether extract, a chloroform extract, an ethyl acetate extract and a n-BuOH extract, respectively. The chloroform extract was subjected to a silica gel CC column using petroleum ether as first eluent and then increasing the polarity with EtOAc, to afford 15 fractions (A—N). Fraction C was further separated by column chromatography with a gradient of CHCl_3_—CH_3_OH to give the title xanthone. The crude product was dissolved in small amount of anhydrous methanol to obtain colourless blocks of (I) by slow evaporation of a methanol solution at 298 K.

### Refinement

H atoms bonded to C atoms were palced in geometrically calculated position and were refined using a riding model, with *U*_iso_(H) = 1.2 *U*_eq_(C). H atoms attached to O atoms were found in a difference Fourier synthesis and were refined using a riding model, with the O—H distances fixed as initially found and with *U*_iso_(H) values set at 1.5 *U*eq(O).

### Crystal data

**Table:**

C_14_H_10_O_4_·H_2_O	*F*(000) = 544
*M**_r_* = 260.24	*D*_x_ = 1.458 Mg m^−^^3^
Monoclinic, *P*2_1_/*c*	Mo *K*α radiation, λ = 0.71073 Å
Hall symbol: -P 2ybc	Cell parameters from 2919 reflections
*a* = 8.8008 (6) Å	θ = 2.1–28.2°
*b* = 7.0856 (4) Å	µ = 0.11 mm^−^^1^
*c* = 19.4596 (9) Å	*T* = 295 K
β = 102.402 (4)°	Block, colourless
*V* = 1185.16 (12) Å^3^	0.38 × 0.26 × 0.24 mm
*Z* = 4	

### Data collection

**Table:**

Bruker SMART CCD diffractometer	2919 independent reflections
Radiation source: fine-focus sealed tube	2269 reflections with *I* > 2σ(*I*)
graphite	*R*_int_ = 0.080
Detector resolution: 0 pixels mm^-1^	θ_max_ = 28.2°, θ_min_ = 2.1°
ω scans	*h* = −6→11
Absorption correction: multi-scan (*SADABS*; Bruker, 1997)	*k* = −8→9
*T*_min_ = 0.959, *T*_max_ = 0.974	*l* = −25→25
8151 measured reflections	

### Refinement

**Table:**

Refinement on *F*^2^	Secondary atom site location: difference Fourier map
Least-squares matrix: full	Hydrogen site location: inferred from neighbouring sites
*R*[*F*^2^ > 2σ(*F*^2^)] = 0.053	H atoms treated by a mixture of independent and constrained refinement
*wR*(*F*^2^) = 0.164	*w* = 1/[σ^2^(*F*_o_^2^) + (0.0823*P*)^2^ + 0.2871*P*] where *P* = (*F*_o_^2^ + 2*F*_c_^2^)/3
*S* = 1.04	(Δ/σ)_max_ < 0.001
2919 reflections	Δρ_max_ = 0.26 e Å^−^^3^
181 parameters	Δρ_min_ = −0.36 e Å^−^^3^
0 restraints	Extinction correction: *SHELXL97* (Sheldrick, 2008), Fc^*^=kFc[1+0.001xFc^2^λ^3^/sin(2θ)]^-1/4^
Primary atom site location: structure-invariant direct methods	Extinction coefficient: 0.095 (8)

### Special details

**Table:**

Geometry. All e.s.d.'s (except the e.s.d. in the dihedral angle between two l.s. planes) are estimated using the full covariance matrix. The cell e.s.d.'s are taken into account individually in the estimation of e.s.d.'s in distances, angles and torsion angles; correlations between e.s.d.'s in cell parameters are only used when they are defined by crystal symmetry. An approximate (isotropic) treatment of cell e.s.d.'s is used for estimating e.s.d.'s involving l.s. planes.
Refinement. Refinement of *F*^2^ against ALL reflections. The weighted *R*-factor *wR* and goodness of fit *S* are based on *F*^2^, conventional *R*-factors *R* are based on *F*, with *F* set to zero for negative *F*^2^. The threshold expression of *F*^2^ > σ(*F*^2^) is used only for calculating *R*-factors(gt) *etc*. and is not relevant to the choice of reflections for refinement. *R*-factors based on *F*^2^ are statistically about twice as large as those based on *F*, and *R*- factors based on ALL data will be even larger.

### Fractional atomic coordinates and isotropic or equivalent isotropic displacement parameters (Å^2^)

**Table:**

	*x*	*y*	*z*	*U*_iso_*/*U*_eq_	
O1	0.65861 (11)	0.14472 (16)	0.26194 (6)	0.0409 (3)	
O2	0.24707 (14)	0.1457 (2)	0.33085 (6)	0.0577 (4)	
O3	0.09211 (11)	0.10250 (16)	0.19163 (6)	0.0412 (3)	
O4	0.11784 (12)	0.12437 (18)	0.05791 (5)	0.0459 (3)	
H4A	0.1386	0.1122	0.0190	0.069*	
O1W	0.16228 (15)	0.0842 (3)	−0.07501 (6)	0.0581 (4)	
H1A	0.181 (3)	0.166 (4)	−0.1024 (17)	0.097 (10)*	
H1B	0.080 (3)	0.039 (4)	−0.0954 (14)	0.079 (8)*	
C1	0.65891 (18)	0.1459 (2)	0.33241 (8)	0.0377 (3)	
C2	0.8053 (2)	0.1522 (2)	0.37783 (9)	0.0491 (4)	
H2A	0.8955	0.1548	0.3602	0.059*	
C3	0.8131 (2)	0.1546 (3)	0.44946 (10)	0.0577 (5)	
H3A	0.9096	0.1575	0.4805	0.069*	
C4	0.6771 (3)	0.1527 (3)	0.47583 (9)	0.0593 (5)	
H4B	0.6837	0.1550	0.5242	0.071*	
C5	0.5341 (2)	0.1475 (2)	0.43070 (9)	0.0497 (4)	
H5A	0.4445	0.1464	0.4487	0.060*	
C6	0.52170 (18)	0.1439 (2)	0.35728 (7)	0.0367 (3)	
C7	0.36878 (17)	0.1408 (2)	0.30878 (7)	0.0360 (3)	
C8	0.37350 (15)	0.13504 (18)	0.23329 (7)	0.0299 (3)	
C9	0.23858 (15)	0.12734 (19)	0.17825 (7)	0.0316 (3)	
C10	0.25162 (16)	0.12938 (19)	0.10794 (7)	0.0343 (3)	
C11	0.39927 (17)	0.1332 (2)	0.09150 (7)	0.0374 (3)	
H11A	0.4075	0.1331	0.0446	0.045*	
C12	0.53188 (17)	0.1372 (2)	0.14382 (8)	0.0372 (3)	
H12A	0.6293	0.1385	0.1325	0.045*	
C13	0.51916 (16)	0.13944 (19)	0.21397 (7)	0.0318 (3)	
C14	0.0097 (2)	0.2739 (3)	0.19948 (11)	0.0578 (5)	
H14A	−0.0904	0.2434	0.2086	0.087*	
H14B	−0.0035	0.3468	0.1570	0.087*	
H14C	0.0681	0.3457	0.2381	0.087*	

### Atomic displacement parameters (Å^2^)

**Table:**

	*U*^11^	*U*^22^	*U*^33^	*U*^12^	*U*^13^	*U*^23^
O1	0.0304 (5)	0.0548 (7)	0.0354 (6)	−0.0022 (4)	0.0027 (4)	−0.0018 (4)
O2	0.0468 (6)	0.0962 (10)	0.0337 (6)	−0.0072 (6)	0.0163 (5)	−0.0084 (6)
O3	0.0315 (5)	0.0527 (7)	0.0412 (6)	−0.0061 (4)	0.0118 (4)	−0.0017 (4)
O4	0.0377 (6)	0.0689 (8)	0.0284 (5)	−0.0012 (5)	0.0010 (4)	0.0009 (4)
O1W	0.0456 (7)	0.0910 (11)	0.0357 (6)	−0.0150 (7)	0.0044 (5)	0.0005 (6)
C1	0.0426 (7)	0.0330 (7)	0.0337 (7)	−0.0023 (5)	0.0000 (6)	−0.0023 (5)
C2	0.0439 (8)	0.0466 (9)	0.0490 (9)	−0.0025 (7)	−0.0071 (7)	−0.0036 (7)
C3	0.0627 (11)	0.0509 (10)	0.0459 (9)	−0.0027 (8)	−0.0187 (8)	−0.0028 (7)
C4	0.0798 (13)	0.0582 (11)	0.0317 (8)	−0.0053 (9)	−0.0063 (8)	−0.0023 (7)
C5	0.0643 (10)	0.0517 (10)	0.0304 (7)	−0.0039 (7)	0.0042 (7)	−0.0028 (6)
C6	0.0453 (8)	0.0339 (7)	0.0285 (7)	−0.0035 (5)	0.0029 (5)	−0.0022 (5)
C7	0.0400 (7)	0.0387 (7)	0.0296 (7)	−0.0042 (5)	0.0082 (5)	−0.0030 (5)
C8	0.0315 (6)	0.0306 (6)	0.0276 (6)	−0.0029 (5)	0.0062 (5)	−0.0006 (5)
C9	0.0307 (6)	0.0333 (7)	0.0315 (7)	−0.0026 (5)	0.0081 (5)	−0.0002 (5)
C10	0.0347 (7)	0.0370 (7)	0.0298 (6)	−0.0018 (5)	0.0039 (5)	0.0014 (5)
C11	0.0415 (7)	0.0442 (8)	0.0286 (7)	−0.0004 (6)	0.0119 (6)	0.0021 (5)
C12	0.0341 (7)	0.0448 (8)	0.0355 (7)	−0.0009 (6)	0.0138 (5)	0.0008 (6)
C13	0.0302 (6)	0.0325 (7)	0.0324 (7)	−0.0016 (5)	0.0055 (5)	−0.0013 (5)
C14	0.0417 (8)	0.0675 (12)	0.0693 (11)	0.0063 (8)	0.0235 (8)	−0.0022 (9)

### Geometric parameters (Å, °)

**Table:**

O1—C1	1.3708 (18)	C4—H4B	0.9300
O1—C13	1.3737 (16)	C5—C6	1.409 (2)
O2—C7	1.2372 (17)	C5—H5A	0.9300
O3—C9	1.3801 (16)	C6—C7	1.469 (2)
O3—C14	1.439 (2)	C7—C8	1.4790 (18)
O4—C10	1.3576 (17)	C8—C13	1.4116 (18)
O4—H4A	0.8200	C8—C9	1.4191 (18)
O1W—H1A	0.83 (3)	C9—C10	1.3973 (19)
O1W—H1B	0.81 (3)	C10—C11	1.4036 (19)
C1—C6	1.394 (2)	C11—C12	1.375 (2)
C1—C2	1.398 (2)	C11—H11A	0.9300
C2—C3	1.381 (3)	C12—C13	1.3935 (19)
C2—H2A	0.9300	C12—H12A	0.9300
C3—C4	1.400 (3)	C14—H14A	0.9600
C3—H3A	0.9300	C14—H14B	0.9600
C4—C5	1.371 (3)	C14—H14C	0.9600
			
C1—O1—C13	119.30 (11)	C13—C8—C9	117.41 (12)
C9—O3—C14	115.13 (12)	C13—C8—C7	118.99 (12)
C10—O4—H4A	109.5	C9—C8—C7	123.59 (12)
H1A—O1W—H1B	104 (3)	O3—C9—C10	117.56 (12)
O1—C1—C6	122.10 (13)	O3—C9—C8	121.77 (12)
O1—C1—C2	115.87 (14)	C10—C9—C8	120.48 (12)
C6—C1—C2	122.02 (14)	O4—C10—C9	117.45 (12)
C3—C2—C1	118.55 (17)	O4—C10—C11	122.66 (13)
C3—C2—H2A	120.7	C9—C10—C11	119.88 (12)
C1—C2—H2A	120.7	C12—C11—C10	120.79 (12)
C2—C3—C4	120.59 (16)	C12—C11—H11A	119.6
C2—C3—H3A	119.7	C10—C11—H11A	119.6
C4—C3—H3A	119.7	C11—C12—C13	119.46 (12)
C5—C4—C3	120.29 (16)	C11—C12—H12A	120.3
C5—C4—H4B	119.9	C13—C12—H12A	120.3
C3—C4—H4B	119.9	O1—C13—C12	114.70 (12)
C4—C5—C6	120.70 (17)	O1—C13—C8	123.34 (12)
C4—C5—H5A	119.6	C12—C13—C8	121.95 (13)
C6—C5—H5A	119.6	O3—C14—H14A	109.5
C1—C6—C5	117.85 (14)	O3—C14—H14B	109.5
C1—C6—C7	121.30 (13)	H14A—C14—H14B	109.5
C5—C6—C7	120.84 (14)	O3—C14—H14C	109.5
O2—C7—C6	121.24 (13)	H14A—C14—H14C	109.5
O2—C7—C8	123.82 (13)	H14B—C14—H14C	109.5
C6—C7—C8	114.92 (12)		
			
C13—O1—C1—C6	−0.65 (19)	C14—O3—C9—C10	−93.42 (16)
C13—O1—C1—C2	−179.73 (12)	C14—O3—C9—C8	91.70 (16)
O1—C1—C2—C3	179.71 (14)	C13—C8—C9—O3	173.03 (12)
C6—C1—C2—C3	0.6 (2)	C7—C8—C9—O3	−7.70 (19)
C1—C2—C3—C4	−0.7 (3)	C13—C8—C9—C10	−1.70 (18)
C2—C3—C4—C5	0.3 (3)	C7—C8—C9—C10	177.57 (12)
C3—C4—C5—C6	0.1 (3)	O3—C9—C10—O4	5.96 (18)
O1—C1—C6—C5	−179.30 (13)	C8—C9—C10—O4	−179.09 (12)
C2—C1—C6—C5	−0.3 (2)	O3—C9—C10—C11	−172.99 (12)
O1—C1—C6—C7	−0.2 (2)	C8—C9—C10—C11	1.96 (19)
C2—C1—C6—C7	178.83 (13)	O4—C10—C11—C12	−179.64 (13)
C4—C5—C6—C1	−0.1 (2)	C9—C10—C11—C12	−0.7 (2)
C4—C5—C6—C7	−179.18 (15)	C10—C11—C12—C13	−0.7 (2)
C1—C6—C7—O2	−177.43 (14)	C1—O1—C13—C12	−179.55 (12)
C5—C6—C7—O2	1.6 (2)	C1—O1—C13—C8	−0.03 (18)
C1—C6—C7—C8	1.57 (19)	C11—C12—C13—O1	−179.57 (12)
C5—C6—C7—C8	−179.36 (13)	C11—C12—C13—C8	0.9 (2)
O2—C7—C8—C13	176.82 (14)	C9—C8—C13—O1	−179.21 (12)
C6—C7—C8—C13	−2.15 (17)	C7—C8—C13—O1	1.49 (19)
O2—C7—C8—C9	−2.4 (2)	C9—C8—C13—C12	0.28 (19)
C6—C7—C8—C9	178.59 (12)	C7—C8—C13—C12	−179.03 (13)

### Hydrogen-bond geometry (Å, °)

**Table:**

*D*—H···*A*	*D*—H	H···*A*	*D*···*A*	*D*—H···*A*
O4—H4A···O1W	0.82	1.90	2.7126 (16)	174
O1W—H1A···O2^i^	0.83 (3)	2.03 (3)	2.857 (2)	174 (3)
O1W—H1B···O4^ii^	0.81 (3)	2.34 (3)	2.9540 (19)	134 (2)
O1W—H1B···O3^ii^	0.81 (3)	2.37 (3)	3.1195 (17)	155 (2)

Symmetry codes: (i) *x*, −*y*+1/2, *z*−1/2; (ii) −*x*, −*y*, −*z*.
